# Cannabis Hyperemesis Syndrome in a Young Patient: A Case Report and Literature Review

**DOI:** 10.7759/cureus.43868

**Published:** 2023-08-21

**Authors:** Noman Khalid, Muhammad Abdullah, Musa Khalil, Muhammad Adil Afzal, Mulham Hindawi

**Affiliations:** 1 Internal Medicine, St. Joseph's University Medical Center, Paterson, USA; 2 Department of Medicine/Department of Public Health and Community Medicine, Shaikh Khalifa Bin Zayed Al-Nahyan Medical and Dental College, Shaikh Zayed Federal Postgraduate Medical Institute at Shaikh Zayed Medical Complex, Lahore, PAK; 3 Medicine, Shaikh Zayed Hospital, Lahore, PAK

**Keywords:** marijuana abuse, cannabis hyperemesis syndrome, cannabis abuse, cannabis legalization, cannabis use

## Abstract

Cannabis hyperemesis syndrome (CHS) is a condition characterized by recurrent episodes of severe vomiting, abdominal pain, and intractable nausea in chronic cannabis users. With the legalization of recreational marijuana in many states, awareness of CHS is crucial to prevent delayed diagnosis or misdiagnosis. This case report presents a 25-year-old male with a history of type 1 diabetes mellitus and chronic cannabis use who presented to the emergency department with vomiting and epigastric pain. Our literature review sheds light on existing treatment options for this syndrome and gives future direction for research.

## Introduction

With the recent legalization of recreational marijuana in several states, individuals now have increased access to cannabis. However, it is important to recognize that cannabis users may not be fully aware of all the side effects associated with its use, including some that are not well-known or well-understood. One such condition is cannabis hyperemesis syndrome (CHS), characterized by recurrent episodes of intractable nausea, abdominal pain, and vomiting lasting 24-48 hours [1]. These symptoms are more common in long-term cannabis users, particularly males. Interestingly, patients with CHS often report using compulsive hot showers to alleviate their symptoms [2]. The pathophysiology of CHS remains unclear, but it can lead to complications such as gastritis, esophagitis, hypokalemia, and acute renal failure [2]. While the definitive treatment for CHS is the cessation of cannabis use, raising awareness about this condition is crucial to prevent delayed diagnosis or misdiagnosis [3].

## Case presentation

A 25-year-old male with a medical history significant for type 1 diabetes mellitus, recurrent admissions for diabetic ketoacidosis (DKA), asthma, hypertension, chronic hepatitis C infection, and cannabis use for six years presented to the emergency department with repetitive vomiting episodes and epigastric pain for the past two days. The vomiting episodes were acute in onset, non-bloody, non-projectile, and initially contained food particles. The patient reported associated severe epigastric abdominal pain, which was localized, rated at 8/10 in intensity, non-radiating, and not relieved by vomiting. The patient acknowledged more than usual cannabis use last night. Multiple hospital admissions for similar symptoms were reported over the past seven months. The patient was taking metoclopramide for probable gastroparesis. The physical examination revealed mild epigastric tenderness without signs of peritoneal irritation. 

Laboratory investigations demonstrated the following findings: sodium (Na^+^) level of 136 mEq/L (normal range: 135-145 mEq/L), chloride (Cl^-^) level of 99 mEq/L (normal range: 96-106 mEq/L), bicarbonate (HCO_3_^-^) level of 20 mEq/L (as part of the basal metabolic panel; normal range: 22-29 mEq/L), lipase level of 18 units/L (normal range: 7-60 units/L), negative acetone, blood glucose level of 377 mg/dL (normal range: 70-99 mg/dL), anion gap of 17 (normal range: 3-11 mEq/L), positive urine toxicology for tetrahydrocannabinol (THC), blood urea nitrogen (BUN) level of 31 mg/dL (normal range: 7-20 mg/dL), creatinine (Cr) level of 1.83 mg/dL (normal range: 0.6-1.2 mg/dL), venous pH of 7.56 (normal range: 7.35-7.45), venous partial pressure of carbon dioxide (PCO_2_) of 23 mmHg (normal range: 35-45 mmHg), and venous bicarbonate level of 20.6 mEq/L (normal range: 22-29 mEq/L).

Computed tomography (CT) scan of the abdomen and pelvis revealed no evidence of bowel obstruction, appendicitis, diverticulitis, or pancreatitis. The abdominal ultrasound did not demonstrate the presence of gallstones. The patient was managed with ketorolac for pain relief, ondansetron for antiemetic effect, pantoprazole for gastrointestinal prophylaxis, insulin therapy, and intravenous fluid resuscitation with 2 liters of fluids. Subsequent laboratory tests showed a declining trend in anion gap and creatinine levels, and the patient's symptoms eventually subsided over the next 24 hours. After ruling out other potential differential diagnoses and considering the persistence of symptoms over the past several months despite prokinetic therapy, and given the relationship of symptoms to heavy cannabis use, a diagnosis of cannabis hyperemesis syndrome was made. The patient received counseling for cannabis use cessation and was discharged two days later.

## Discussion

Cannabis hyperemesis syndrome (CHS) is characterized by the sudden onset of severe vomiting in cannabis users that often does not respond to conventional antiemetic therapies. This condition is typically observed in individuals who have been using cannabis chronically for at least six months. Patients experience recurrent episodes of vomiting, and the prevalence of CHS has increased since its first report in 2004, possibly due to the widespread use of cannabis following its legalization for medical and recreational purposes in many US states [4].

Understanding the underlying pathophysiology of CHS is crucial for developing effective management strategies and optimizing resource utilization in hospital emergency settings. This requires a deeper understanding of the human body's endocannabinoid system, including its ligands, signalling pathways, and receptors [5]. THC, the active component in cannabis, is commonly used in chemotherapy patients to alleviate nausea and vomiting [6]. THC exerts its antiemetic effects by interacting with various receptors in the gastrointestinal system. However, chronic cannabis users can experience something paradoxical to the antiemetic properties of THC, known as CHS.

The pathophysiology of CHS remains poorly understood, with theories suggesting that chronic overstimulation of endocannabinoid receptors disrupts the body's control over vomiting [7]. Cannabis has a biphasic effect, initially acting as an antiemetic but potentially causing hyperemesis at higher doses and prolonged use. Therefore, early recognition of CHS is essential in preventing volume depletion.

**Figure 1:**
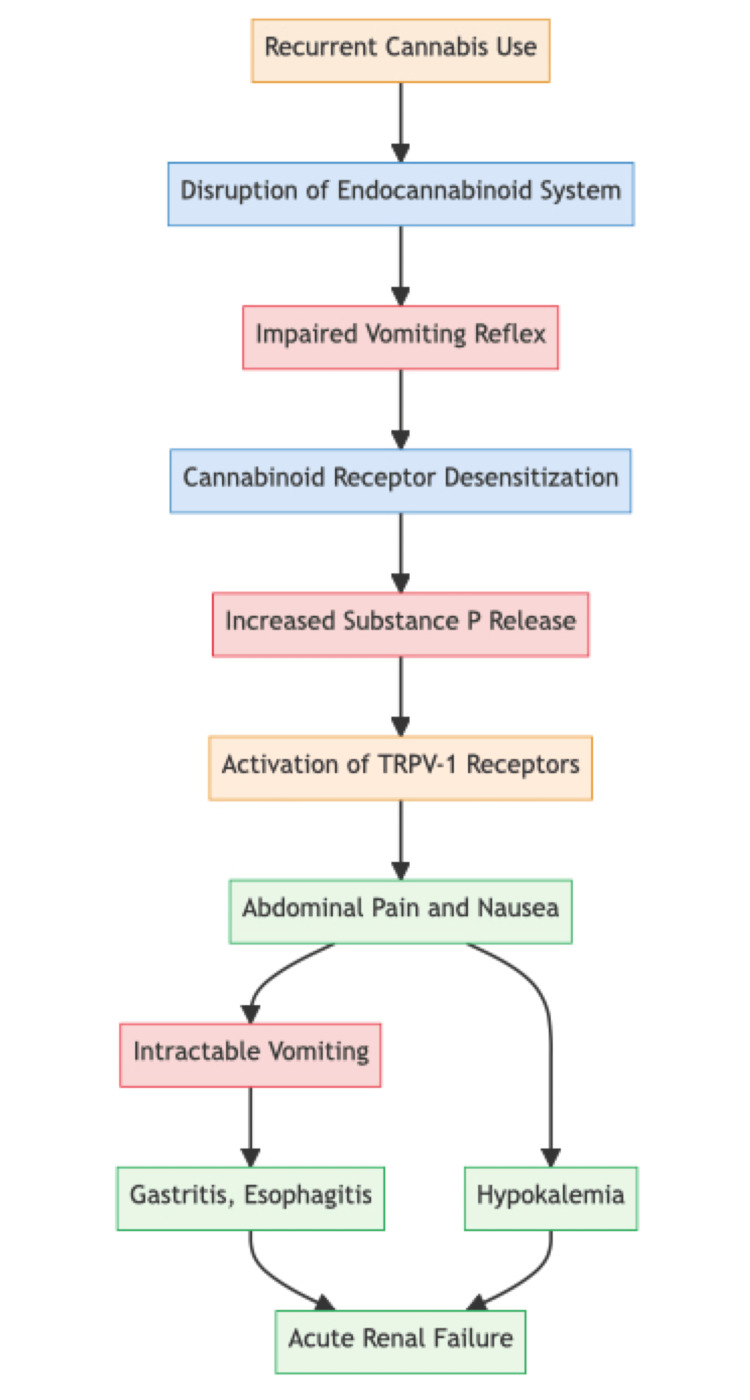
Pathophysiology of Cannabis Hyperemesis Syndrome TRPV-1: Transient receptor potential vanilloid 1

CHS occurs in three phases: the prodromal phase, the hyperemesis phase, and the postdrome phase. The prodromal phase, which can last for months, is characterized by abdominal discomfort, anorexia, and nausea. However, patients can maintain their usual eating habits during this phase. In the hyperemesis phase, patients experience repetitive vomiting, similar to cyclical vomiting syndrome. The acute vomiting phase usually lasts one to two days, while abdominal pain, spanning the umbilical and periumbilical areas, may persist for around 10 days. It is during this phase that patients seek medical care due to bothersome symptoms and where most complications of CHS develop. The last phase occurs when patients stop using cannabis, but CHS can recur if cannabis use is resumed [8]. The pathophysiology and natural history of CHS are described in Figure 1.

Since acute bouts of vomiting are the main feature of CHS, it is essential to rule out other pathologies that present with similar symptoms. Distinguishing CHS from conditions like diabetic ketoacidosis (DKA), cyclical vomiting syndrome, bulimia nervosa, viral gastroenteritis, gastroparesis, and labyrinthitis can be challenging. A patient's history of chronic cannabis use is crucial for the diagnosis. Although CHS is considered a variant of cyclical vomiting syndrome, early recognition remains essential due to the potential development of acute kidney injury, rhabdomyolysis, and Mallory-Weiss syndrome in CHS [7]. Sometimes it’s challenging to differentiate between DKA and CHS because of overlapping symptoms. In patients with ketosis and suspected DKA, atypically high PH and bicarbonate levels and a history of cannabis point more towards hyperglycemic ketosis due to CHS rather than DKA [9]. Thus, in adults with type 1 diabetes presenting with symptoms of DKA, but atypical labs, a history of cannabis use should be taken.

Currently, the Rome IV criteria are widely used for the early recognition of CHS. However, specific parameters such as the duration of chronic cannabis use and the cessation of cannabis use need further definition [5]. Compulsive hot bathing can serve as a vague indicator of CHS. External stressors such as cold have been linked to exacerbations of CHS, while hot showers provide relief by activating transient receptor potential vanilloid 1 (TRPV-1) receptors, similar to the effects of capsaicin [7]. However, compulsive hot bathing is neither specific nor sensitive as an indicator of CHS.

The literature review revealed that the cases of Cannabis Hyperemesis Syndrome (CHS) predominantly involved male individuals aged 25 to 40 years, with only one reported case involving a female. Notably, the duration of cannabis use among these cases exhibited a wide range, spanning from 4 years to 27 years, but no case was reported with less than four years of use, indicating the chronic nature of the condition. The duration of CHS symptoms varied from one day to seven years. CHS treatment approaches encompassed a spectrum of interventions, ranging from symptom resolution upon cessation of cannabis use or hot showers to cases necessitating comprehensive medical management. Further details of the literature review findings can be found in Table 1.

**Table 1:** Case Reports of Cannabis Hyperemesis Syndrome in the Literature

Author, year	Age (years), Sex	Frequency of use	Duration of use	Symptoms	Duration of symptoms	Symptom resolution
Present case	25, M	Daily	Six Years	Repetitive vomiting episodes and epigastric pain	Seven months	Ketorolac, Ondansetron, Pantoprazole, Insulin, IV fluids
Patel et al., 2023 [10]	28, M	-	-	Flank pain, nausea, vomiting	One day	Normal saline bolus, Dilaudid, Tamsulosin, Ceftriaxone
Le Sala et al., 2022 [11]	40, F	Daily	27 years	Diffused non-localized abdominal pain, nausea, intractable non-bloody vomiting	-	Ondansetron, Prochlorperazine, Promethazine, Metoclopramide, Haloperidol,
Kuzin et al., 2021 [12]	29, M	-	-	Vomiting episodes, stomach cramps and headache, diarrhea	Seven years (diagnosed Seven years ago)	Pantoprazole, Quetiapine, Haloperidol, Lorazepam, Metamizole, Itinerol B6, Caffeine, Pyridoxine, Paracetamol, Ondansetron, Riopan stomach Gel®, fluid infusion
Howard I, 2019 [13]	31, M	Thrice a week	Four years	Abdominal pain, nausea, and vomiting of two months duration	Two months	Cessation of cannabis use
Mahmad et al., 2015 [14]	32, M	-	19 years	Nausea, vomiting, abdominal pain	Five days	Hot showers
Warner et al., 2014 [15]	28, M	Daily	13 years	Vomiting, abdominal pain	-	Hot showers

The definitive treatment for CHS is the cessation of cannabis use, although haloperidol may be used to abort severe hyperemesis episodes [16]. Various management strategies are currently employed for CHS, but definitive first and second-line options are yet to be established. Some treatment options include antiemetics, aprepitant, haloperidol, ondansetron, droperidol, propranolol, benzodiazepines, capsaicin, and compulsive hot bathing. Aprepitant, a Neurokinin-1 antagonist, has shown promise in treating CHS by regulating substance P and reducing nausea and vomiting [17,18]. Providing guidance to patients regarding the cessation of cannabis use holds a significant place in the management of Cannabis Hyperemesis Syndrome (CHS). Initially, patients might exhibit reluctance to attribute their symptoms to cannabis consumption; therefore, physicians should adopt an empathetic approach to foster understanding. Given the absence of effective pharmacological interventions for cannabis cessation, strategies such as motivational enhancement treatment (MET), cognitive behavioral therapy (CBT), and contingency management (CM) become pivotal in addressing this aspect of care.

## Conclusions

In conclusion, as cannabis use expands, healthcare professionals must be vigilant in recognizing and addressing potential side effects like CHS. Education and awareness are crucial for the well-being of cannabis users and providing appropriate care for those affected by this syndrome. The lack of established first and second-line treatment options highlights the need for further research in this area.
